# Traffic noise in the bedroom in association with markers of obesity: a cross-sectional study and mediation analysis of the respiratory health in Northern Europe cohort

**DOI:** 10.1186/s12889-023-16128-2

**Published:** 2023-06-27

**Authors:** Triin Veber, Andrei Pyko, Hanne Krage Carlsen, Mathias Holm, Thorarinn Gislason, Christer Janson, Ane Johannessen, Johan Nilsson Sommar, Lars Modig, Eva Lindberg, Vivi Schlünssen, Karolin Toompere, Hans Orru

**Affiliations:** 1 Institute of Family Medicine and Public Health, University of Tartu, Tartu, Estonia; 2 Centre for Occupational and Environmental Medicine, Region Stockholm, Stockholm, Sweden; 3 Institute of Environmental Medicine, Karolinska Institute, Stockholm, Sweden; 4 Department of Occupational and Environmental Medicine, School of Public Health and Community Medicine, Institute of medicine, Sahlgrenska Academy, University of Gothenburg, Gothenburg, Sweden; 5 Faculty of Medicine, University of Iceland, Reykjavik, Iceland; 6 Department of Medical Sciences: Respiratory, Allergy and Sleep Research, Uppsala University, Uppsala, Sweden; 7 Department of Global Public Health and Primary Care, University of Bergen, Bergen, Norway; 8 Section of Sustainable Health, Department of Public Health and Clinical Medicine, Umeå University, Umeå, Sweden; 9 Research Unit for Environment, Occupation and Health, Danish Ramazzini Centre, Aarhus University, Aarhus, Denmark

**Keywords:** Noise, Obesity, Overweight, Adiposity, Indoor, Nocturnal, Self-reported, Road Traffic

## Abstract

**Background:**

Previous research suggests an association between road traffic noise and obesity, but current evidence is inconclusive. The aim of this study was to assess the association between nocturnal noise exposure and markers of obesity and to assess whether sleep disturbance might be a mediator in this association.

**Methods:**

We applied data from the Respiratory Health in Northern Europe (RHINE) cohort. We used self-measured waist circumference (WC) and body mass index (BMI) as outcome values. Noise exposure was assessed as perceived traffic noise in the bedroom and/or the bedroom window’s location towards the street. We applied adjusted linear, and logistic regression models, evaluated effect modifications and conducted mediation analysis.

**Results:**

Based on fully adjusted models we found that women, who reported very high traffic noise levels in bedroom, had 1.30 (95% CI 0.24–2.37) kg/m^2^ higher BMI and 3.30 (95% CI 0.39–6.20) cm higher WC compared to women, who reported no traffic noise in the bedroom. Women who reported higher exposure to road traffic noise had statistically significant higher odds of being overweight and have abdominal obesity with OR varying from 1.15 to 1.26 compared to women, who reported no traffic noise in the bedroom. For men, the associations were rather opposite, although mostly statistically insignificant. Furthermore, men, who reported much or very much traffic noise in the bedroom, had a statistically significantly lower risk of abdominal obesity. Sleep disturbance fully or partially mediated the association between noise in bedroom and obesity markers among women.

**Conclusion:**

Our results suggest that self-reported traffic noise in the bedroom may be associated to being overweight or obese trough sleep disturbance among women, but associations were inconclusive among men.

**Supplementary Information:**

The online version contains supplementary material available at 10.1186/s12889-023-16128-2.

## Background

Environmental noise is one of the most important environmental stressors impacting health ([1, 2]. The main source of environmental noise in Europe is road traffic, which is a growing environmental concern due to urbanization and increasing traffic volumes in cities world-wide [3, 4]. Exposure to traffic noise has been linked to quality of life and wellbeing [5], mental health [6], sleep disturbance [7] as well as cardiovascular and metabolic health, including being overweight or obese [8–11].

Recently, being overweight or obese has received great attention in public health as cases are rapidly increasing worldwide [12]. Obesity is one of the main risk factors contributing to the global burden of disease [13]. Most of the deaths related to obesity, measured as increased waist circumference (WC) or elevated body mass index (BMI), result as cardiovascular disease; however, several studies suggest that obesity is a risk factor also for diabetes, cancer, and for many other non-communicable diseases [12, 14]. Well-recognized risk factors for obesity are behavioral risk factors, like physical inactivity and a high-calorie diet [15], but so far, little is known about the role of environmental risk factors.

Besner et al. (2014) have posited that road traffic noise may contribute to increased risk of obesity/being overweight through stress reactions. Traffic noise can induce acute stress with release of stress hormones depending on an individual’s reaction. If they respond through the active strategy “fight or flight”, the response involves activation of the sympathetic–adrenal–medullar axis and catecholamine release. The response through the repressed strategy “defeat” associated with distress, anxiety, etc. involves hypothalamic–pituitary–adrenocortical (HPA) activation and cortisol release [16, 17] HPA activity dysregulation has been reported to contribute to obesity and other metabolic complications [18] Moreover, stress reactions can be mediated by sleep disturbance [19] During sleep, the brain is still able to perceive, evaluate and react to environmental sounds, and the noise levels that cause stress reactions are much lower during sleep. A stress response during sleep has been observed from very low noise levels as L_Amax_ 33 dB [20]. Traffic noise in the bedroom can also lead to sleep deprivation [19]. Sleep deprivation affects appetite and energy expenditure regulating hormones [21] Numerous epidemiological studies link sleep deprivation with obesity [22].

Exposure to traffic noise has been increasingly reported to be associated with markers of obesity, but the evidence is still limited and the results are rather controversial [11, 23–26]. In all studies, included in the systematic review conducted by van Kempen et al. (2018), increased traffic noise was associated with increases in obesity markers, but the evidence was evaluated to be “low”, because of the limited number of studies available in this topic. Another systematic review suggested that noise from different sources (transportation, leisure, occupational) tends to be positively associated with waist circumference, but not with BMI [25]. Wallas et al. (2019), found an association between road traffic noise and BMI among school-age children. A Swedish cohort study found an association between residential levels of road traffic noise and WC [23]. Moreover, some groups of people seem to be more vulnerable, as in a Norwegian cross-sectional study, no associations with WC or BMI were found in the total population, but associations were statistically significant among highly noise-sensitive women [26].

In most of the epidemiological studies, the modelled noise levels at the façade have been applied; however, individual actual noise exposure depends on numerous factors, including the floor of the dwelling, façade insulation, bedroom location, hours spent at home, etc. [27]. Many traffic noise models have been developed not considering all factors like different seasons, different days of week, different conditions (like dry and wet surface) of road etc. [28]. Therefore, studies that use outdoor modelled noise levels as the exposure variable, can under- or overestimate the individual noise exposure. Indoor nocturnal noise measured with a noise dosimeter in participants’ bedrooms during a week was associated with BMI in women but not in men in Taiwan [29]. Huang et al. [30] combined self-reported noise perception with data from noise-monitoring stations and found road traffic noise to be associated with all of the metabolic syndrome’s components, including abdominal obesity in a cohort study in Taiwan. Nevertheless, evidence is very limited about indoor nocturnal road traffic noise effects on obesity and being overweight.

The aim of the present study was to assess the association between self-reported nocturnal noise exposure and self-measured markers of overweight/obesity.

## Methods

### Study population

We applied data collected through self-administrated questionnaires and self-measurements from the cohort study Respiratory Health in Northern Europe (RHINE) III; although, this analysis has a cross-sectional design. The RHINE was initiated in 1989–1992, with 3000–4000 randomly selected men and women 17–45 years of age from each of the seven study centers: Reykjavik, Iceland; Bergen, Norway; Umeå, Uppsala and Gothenburg, Sweden; Aarhus, Denmark and Tartu, Estonia. RHINE III (after 20 years follow-up) was conducted in 2010–2012 and a total of 13,578 people in age 38–66 years (mean 51.6, SD 7.2) responded to the questionnaire [31]. 61% of the original respondents participated in RHINE III [31]. The participants received a measuring tape and completed a questionnaire covering their health, lifestyle, indoor and outdoor environment, family history, socio-economic factors, sleep disturbance, noise hearing in participants’ bedrooms, and the bedrooms’ location toward the street.

We included all participants for whom the necessary data were available for the analysis (in total 13,146). We excluded people who reported unrealistic waist circumferences, height, and weight data. People who reported that they spend less than 5 h at home per day were also left out as this would cause exposure misclassification.

### Noise exposure assessment

Participants were asked to evaluate noise exposure in their bedroom through the two questions:

In your bedroom, can you hear traffic noise?

Answer alternatives: (1) not at all, (2) a little, (3) a great deal, and (4) very much

Does your bedroom window face a nearby street (< 20 m)?

Answer alternatives: (1) no, (2) yes, a street with little traffic, (3) yes, a street with moderate traffic, and (4) yes, a street with a great deal of traffic.

Different categorizations of noise exposure were used. For question 1, about traffic noise hearing in the bedroom, four categories (not at all, a little, a great deal, very much) and a binary variable (no /yes) were used. In the binary variable, participants who answered “not at all” were categorized as “no” and those who answered “a little, a great deal or very much” were categorized as “yes.” For question 2: “Does your bedroom window face a nearby street (< 20 m)?” four categories (no, a street with little traffic, a street with moderate traffic, a street with a great deal of traffic) and a binary variable (no/yes) were used. In addition, we composed a combined indicator. In the combined indicator, we considered being exposed only those who reported ‘yes’ to both – bedroom window location toward a street and traffic noise hearing in the bedroom. Not exposed were considered participants who had either ‘no’ window towards the street and/or ‘no’ traffic noise in bedroom.

### Assessment of outcome

The assessment of outcomes was based on self-reported data in the RHINE III questionnaire. As a marker of abdominal obesity, we used WC. A measuring tape was sent to the participants together with the questionnaire, and they were asked to measure their waist at the level of the navel, while standing and under the clothes. We applied World Health Organization (WHO) suggested cut-off value to define abdominal obesity: ≥102 cm for men and ≥ 88 cm for women (WHO, 2011b). As a marker of general obesity, we used BMI, calculated as weight (kg) divided by height (m^2^) with a cutoff at BMI ≥ 25 to define being overweight and BMI ≥ 30 to define obesity.

### Covariates

Potential confounders were chosen based on literature and using the software DAGitty (www.dagitty.net). To estimate the minimal adjustment set, and better visualize the different possible confounders, we developed a Directed Acyclic Graph (DAG) (Fig. S1). According to software DAGitty, minimal sufficient adjustment sets for estimating the total effect of noise hearing in the bedroom on obesity/being overweight were: (1) hours spent at home; study center, type of accommodation; or (2) study center; type of accommodation; age; working status. Based on minimal sufficient adjustment set, suggested by DAGitty, we run Model 1 adjusted for sex, age (continuous), study center, working status and type of accommodation.

The fully adjusted model (Model 2) included: sex, age (continuous), study center, smoking status (current, former, never), marital status (single, married or cohabitating, divorced or widowed), working status (no, yes), education (primary school, secondary or technical school, university or college), type of accommodation (detached house, semidetached or terraced house, apartment), hours spent at home (continuous), exercise level (less than once a week, once a week, 2–3 times a week, daily), having children (no, yes) and family history of obesity (no, yes).We included family history of obesity into the main model as a surrogate measure for genetic inheritance and familial disposition. Family history of obesity was evaluated by the question “What picture best describes the body shape of each of your biological parents at age 50 years?” Participants could choose between body shape pictures numbered 1 to 9. Body silhouettes as a tool to reflect obesity in the past are described by Lønnebotn et al. (2018). When one or both biological parents’ body silhouettes were marked 5 or upwards, the participant was considered to have a family history of obesity [32].

We also investigated how the associations are affected by sex, physical activity, air pollution, study centers, type of accommodation, hours spent at home, and residential history. As the air pollution indicator, we used annual average concentrations of nitrogen oxides (NO_X_) for the years 2009–2011 modelled for the participants’ home addresses from a previous study of the same cohort [33]. In stratified analysis we used NO_X_ in tertiles: low (≤ 8.18 µg/m^3^), medium (8.19–10.69 µg/m^3^) and high (≥ 10.7 µg/m^3^). We had air pollution data for 9655 participants. Participants were divided into two groups by residential history: (1) the same address for 10 years, and (2) the address had changed over 10 years.

A sleep disturbance score was calculated using sleep and daytime insomnia symptoms reported during last months based on the next questions:

“How often has it occurred in the last months:

1. …that you have difficulty in getting to sleep at night?
2. …that you wake up repeatedly during the night?
3. …that you feel drowsy in the daytime?
4. …that you wake up too early and have difficulty in getting to sleep again?”

Answers were coded: 1: Never or almost never or less than once a week; 2: Once or twice a week; 3: 3–5 nights/days a week; 4: Almost every day or night. For each respondent, all answers for the all 4 questions (scores 1 to 4, respectively) were summed to obtain sleep disturbance score ranging from 4 to 16. Sleep disturbance score was then divided into 4 levels: Level 1 (Score 4, answered to the all questions never or less than once a week); Level 2 (scores 5 to 6); Level 3 (scores 7 to 8); Level 4 (scores 9 and more).

### Statistical analyses

Differences in descriptive statistics were assessed for continuous variables, with a T-test or Mann-Whitney U-test, and for categorical variables a Pearson’s χ^2^ test. Bonferroni correction was used for multiple comparisons. To analyze the relationship between noise exposure and the continuous outcomes WC and BMI, linear regression models were used. Binary outcomes (being overweight, obesity, and central obesity) were analyzed using logistic regression models. The mediation effect was tested using non-parametric bootstrapping by PROCESS macro version 4.2 model 4 in SPSS [34]. The number of bootstrap samples in the analysis was 5000.

We first ran the models with adjustment for minimal sufficient adjustment set suggested by DAGitty software (Model 1). Then, we ran regression models with adjustment for all confounders (Model 2). A p-value of less than 0.05 was considered statistically significant. We added interaction terms to the models to estimate possible effect modification with sex, physical activity, air pollution group, study center residential history, type of accommodation and hours spent at home. The interactions were tested using the F-test and p-value of 0.1 was used as significance level.

The assumptions of the linear regression models were tested using normal probability plots and scatter plots of residuals against the predicted values (homoscedasticity). The relationships between continuous variables and obesity markers were checked for linearity. Multicollinearity was assessed using the variance inflation factor (VIF). A VIF > 5 was considered an indication of the presence of multicollinearity. Statistical analyses were performed using IBM SPSS Statistics for Windows, Version 28.0 (Armonk, NY IBM Corp).

## Results

In total, 13,096 participants answered the question *“In your bedroom, can you hear traffic noise?*” and 13,088 participants answered the question “*Does your bedroom window face a nearby street (< 20 m)?”.* Half of the participants (51.5%) reported that they can hear traffic noise in their bedroom. The highest proportion of participants who reported that they can hear traffic noise in their bedroom was in Tartu (72.9%) and lowest was in Gothenburg (38.9%). Altogether 42.6% of the participants reported that their bedroom window faces a nearby street closer than 20 m, with the highest proportion in Reykjavik (63.6%) and the lowest in Aarhus (34.1%).

The mean BMI was 26.0 (SD 4.5) kg/m^2^, and it varied between different study centers. The highest mean BMI was in Reykjavik (27.2, SD 4.6) kg/m^2^ and the lowest in Aarhus (25.2, SD 4.6) kg/m^2^ (Fig. S2). The mean waist circumference was 87.2 (SD 12.3) cm among women and 97.5 (SD 11.1) cm among men (Fig. S3 and S4). WC was significantly different by study centers – being the highest among women in Gothenburg (89.9, SD 12.2 cm) and lowest in Aarhus (83.9, SD 11.9 cm). The highest WCs among men were in Reykjavik (100.3, SD 12.3 cm) and in Tartu (100.2, SD 13.2 cm) and the lowest in Aarhus (93.6, SD 9.8 cm). The prevalence of being overweight was 54.9% (lowest in Aarhus 45.7% and highest in Reykjavik 66.3%), and the prevalence of obesity was 15.3% in the study population. The prevalence of abdominal obesity was 44.6% among women and 31.1% among men, and it varied from 31.9% in Aarhus to 54.0% in Gothenburg for women and from 16.9% in Aarhus to 42.1% in Reykjavik for men.

There were some statistically significant differences in the study population characteristics according to noise exposure (Table 1). People who reported higher noise levels in their bedroom and/or had their bedroom window facing the street were more likely women, current smokers, less educated, unemployed, single or divorced, without children, with lower physical activity, lived in apartments, and spent more time at home.

**Table 1:** Descriptive characteristics of the study population in relation to self-reported traffic noise in bedroom. The number of participants in each group with percentages in brackets and mean values with+- standard deviation are reported

	In your bedroom, can you hear traffic noise?		Does your bedroom window face a nearby street (< 20 m)?	
	No	Yes	No	Yes
**Age (years)**	51.7 (± 7.3)	51.5 (± 7.2)	51.6 (± 7.2)	51.6 (± 7.2)
**Sex**				
Women	3255 (51.3)	3680 (54.5)	3900 (52.0)	3029 (54.3)
Men	3091 (48.7)	3070 (45.5)	3607 (48.0)	2552 (45.7)
**Smoking status**				
Current	739 (11.8)	912 (13.9)	893 (12.2)	758 (13.9)
Former	2436 (39.0)	2638 (40.3)	2878 (39.3)	2194 (40.2)
Never	3073 (49.2)	2989 (45.7)	3551 (48.5)	2504 (45.9)
**Education**				
Primary school	704 (11.1)	775 (11.6)	793 (10.6)	681 (12.3)
Secondary or technical school	2566 (40.6)	2867 (42.7)	3134 (42.0)	2298 (41.4)
University or college	3044 (48.2)	3067 (45.7)	3540 (47.4)	2568 (46.3)
**Working status**				
No	785 (12.5)	1043 (15.6)	1013 (13.6)	811 (14.6)
Yes	5519 (87.5)	5650 (84.4)	6438 (86.4)	4727(85.4)
**Type of accommodation**				
Detached house	3520 (56.7)	3093 (46.6)	4128 (56.2)	2487 (45.3)
Semidetached or terraced house	1065 (17.1)	1047 (15.8)	1224 (16.7)	887 (16.2)
Apartment	1627 (26.2)	2498 (37.6)	1999 (27.2)	2118 (38.6)
**Marital status**				
Single	537 (8.5)	817 (12.3)	708 (9.5)	644 (11.7)
Married or cohabitating	5173 (82.4)	5034 (75.6)	5994 (80.6)	4206 (76.3)
Divorced or widowed	571 (9.1)	808 (12.1)	717 (9.7)	662 (12.0)
**Parent obesity**				
No	1310 (22.9)	1365 (22.6)	1563 (23.1)	1110 (22.3)
Yes	4416 (77.1)	4669 (77.4)	5216 (76.9)	3864 (77.7)
**Children**				
No children	745 (11.8)	1002(14.9)	963 (12.9)	782 (14.1)
One or more children	5576 (88.2)	5718 (85.1)	6513 (87.1)	4776 (85.9)
**Level of exercise**				
Less than once a week	1406 (22.3)	1495 (22.5)	1606 (21.6)	1289 (23.4)
Once a week	1156 (18.4)	1197 (18.0)	1335 (18.0)	1017 (18.5)
2–3 times a week	2405 (38.2)	2458 (37.0)	2813 (37.9)	2048 (37.2)
Daily	1325 (21.1)	1495 (22.5)	1669 (22.5)	1151 (20.9)
**Mean hours spent at home**	13.7 (± 3.2)	13.9 (± 3.4)	13.7 (± 3.3)	13.9 (± 3.2)
**Sleep disturbance**				
Level 1^a^	1060 (29.0)	1087 (25.1)	1265 (28.4)	882 (25.0)
Level 2^b^	1125 (30.8)	1325 (30.6)	1349 (30.3)	1101 (31.2)
Level 3^c^	749 (20.5)	889 (20.5)	9911 (20.4)	727 (20.6)
Level 4^d^	722 (19.7)	1034 (23.9)	932 (20.9)	824 (23.3)
**Waist circumference men**	97.6 (± 11.0)	97.3 (± 11.2)	97.2 (± 11.0)	97.8 (± 11.2)
**Waist circumference women**	86.9 (± 11.9)	87.5 (± 12.7)	86.8 (± 12.1)	87.8 (± 12.5)
**BMI**	25.9 (± 4.3)	26.1 (± 4.7)	25.9 (± 4.4)	26.2 (± 4.6)
**Obesity**				
No	5414 (85.7)	5636 (83.7)	6399 (85.5)	4647 (83.6)
Yes	906 (14.3)	1095 (16.3)	1083 (14.5)	914 (16.4)
**Overweight**				
No	2868 (45.7)	2969 (44.5)	3423 (46.2)	2411 (43.7)
Yes	3403 (54.3)	3696 (55.5)	3984 (53.8)	3110 (56.3)
**Abdominal obesity men (WC > 102)**				
No	1937 (67.9)	1990 (70.0)	2319 (69.3)	1609 (68.5)
Yes	916 (32.1)	858 (30.0)	1026 (30.7)	714 (31.5)
**Abdominal obesity women (WC > 88)**				
No	1697 (56.6)	1859 (54.1)	2075 (57.1)	1478 (52.9)
Yes	1300 (43.4)	1578 (45.9)	1560 (43.3)	1315 (47.1)

^a^Sleep score 4, ^b^Sleep score 5–6, ^c^Sleep score 7–8, ^d^Sleep score ≥ 9

We found statistically significant positive associations in models with minimal adjustment set (Model 1) between self-reported traffic noise in the bedroom and the BMI in the total population (Table 2).

**Table 2:** Associations between self-reported noise in bedroom and continuous obesity markers in total population

	Model	BMI, kg/m^2^		WC, cm	
		N	Slope (= beta) (95% CI)	N	Slope (= beta) (95% CI)
In your bedroom, can you hear traffic noise? (no/ yes)	Model 1	12,518	0.19 (0.04 to 0.34)*	11,629	0.39 (-0.03 to 0.82)
	Model 2	10,472	0.12 (-0.04 to 0.28)	9,795	0.22 (-0.22 to 0.66)
Does your bedroom window face a nearby street (< 20 m)? (no/yes)	Model 1	12,512	0.13 (-0.02 to 0.28)	11,625	0.26 (-0.16 to 0.69)
	Model 2	10,464	0.08 (-0.081 to 0.24)	9789	0.15 (-0.30 to 0.59)
Participant reported at the same time bedroom window towards street and traffic noise hearing (no/yes)	Model 1	12,500	0.20 (0.04 to 0.36)*	11,615	0.34 (-0.11 to 0.80)
	Model 2	10,457	0.13 (-0.04 to 0.30)	9783	0.16 (-0.31 to 0.64)

*p < 0.05. Model 1 is adjusted for sex, age (continuous), study center, working status and type of accommodation. Fully adjusted model 2 is adjusted for sex, age (continuous), study center, smoking status, marital status, working status, education, type of accommodation, hours spent at home (continuous), having children, family history of obesity and exercise level

We found a significant effect modification by sex in associations between traffic noise hearing in bedroom and BMI (p = 0.009), and WC (p = 0.007). The positive associations were statistically significant mainly for women. Women who reported that they could hear very much traffic noise in their bedroom had on average 1.30 (95% CI 0.24 to 2.37) kg/m^2^ higher BMI and 3.30 (95% CI 0.39 to 6.20) cm larger WC compared to women who could not hear the traffic noise in the bedroom based on fully adjusted model 2 (Table 3). For men no associations and an opposite statistically significant association were found – men who could hear much traffic noise in their bedroom had smaller WC. We also identified an exposure-response association – the higher noise hearing corresponded to higher beta values for women and lower values for men (Table 3).

**Table 3:** Associations between self-reported traffic noise in bedroom and continuous obesity markers stratified by sex

			BMI, kg/m^2^		WC, cm	
			N	Slope (= beta) (95% CI)	N	Slope (= beta) (95% CI)
In your bedroom, can you hear traffic noise?	**M1** ^**a**^	**Women** *(ref no)*	3123		2880	
		Little	3057	0.33 (0.10 to 0.57)*	2876	0.85 (0.22 to 1.48)*
		Much	385	0.42 (-0.07 to 0.91)	385	1.28 (-0.06 to 2.62)
		Very much	85	1.46 (0.47 to 2.45)*	75	5.69 (2.93 to 8.45)*
		**Men** *(ref no)*	2849		2722	
		Little	2583	0.02 (-0.19 to 0.22)	2408	-0.17 (-0.76 to 0.43)
		Much	289	-0.23 (-0.69 to 0.24)	265	-1.56 (-2.92 to -0.20)*
		Very much	48	-0.59 (-1.67 to 0.49)	46	-1.63 (-4.73 to 1.47)
	**M2** ^**b**^	**Women** *(ref no)*	2616		2439	
		Little	2487	0.30 (0.05 to 0.54)*	2350	0.77 (0.11 to 1.43) *
		Much	309	0.42 (-0.01 to 0.94)	287	1.36 (-0.70 to 2.78)
		Very much	67	1.30 (0.24 to 2.37)*	62	3.30 (0.39 to 6.20)*
		**Men** *(ref no)*	2536		2357	
		Little	2187	-0.09 (-0.30 to 0.13)	2050	-0.41 (-1.02 to 0.20)
		Much	230	-0.30 (-0.80 to 0.19)	211	-1.77 (-3.23 to -0.32)*
		Very much	41	-0.76(-1.90 to 0.35)	40	-2.04 (-5.23 to 1.16)
Does your bedroom window face a nearby street (< 20 m)? Toward street with …	**M1**	**Women** *(ref no)*	3722		3481	
		…little traffic	2198	0.15 (-0.09 to 0.40)	2036	0.29 (-0.37 to 0.95)
		… moderate traffic	530	0.48 (0.06 to 0.90)*	492	1.34 (0.19 to 2.48)*
		… much traffic	196	0.57 (-0.10 to 1.23)	176	2.30 (0.47 to 4.13)*
		**Men** *(ref no)*	3427		3186	
		…little traffic	1880	0.07 (-15 to 0.29)	1746	0.10 (-0.53 to 0.74)
		…moderate traffic	426	-0.01 (-0.40 to 0.37)	384	-0.72 (-1.85 to 0.42)
		…much traffic	134	-0.56 (-1.21 to 0.10)	125	-1.52 (-3.43 to 0.40)
	**M2**	**Women** *(ref no)*	3087		2907	
		…little traffic	1801	0.08 (-0.17 to 0.34)	1679	0.07 (-0.63 to 0.76)
		… moderate traffic	435	0.37 (-0.08 to 0.81)	409	1.2 (-0.08 to 2.32)
		… much traffic	152	0.45 (-0.27 to 1.17)	140	1.53 (-0.43 to3.49)
		**Men** *(ref no)*	2916		2724	
		…little traffic	1605	0.03 (-0.20 to 0.26)	1507	0.13 (-0.52 to 0.79)
		… moderate traffic	358	-0.08 (-0.48 to 0.33)	320	-0.79 (-1.98 to 0.41)
		… much traffic	111	-0.40 (-1.10 to 0.29)	104	-1.62 (-3.62 to 0.38)
Participant reported at the same time bedroom window towards street and traffic noise hearing	**M1**	**Women** *(ref no)*	4523		1978	
		Yes	2116	0.37 (0.14 to 0.61)*	4201	0.89 (0.24 to 1.53)*
		**Men** *(ref no)*	4167		3866	
		Yes	1695	0.01 (-0.21 to 0.22)	1571	-0.31 (-0.93 to 0.32)
	**M2**	**Women** *(ref no)*	3753		3517	
		Yes	1717	0.28 (0.03 to 0.53)*	1613	0.64 (-0.03 to 1.33)
		**Men** *(ref no)*	3566		3327	
		Yes	1422	0.05 (-0.28 to 0.18)	1327	-0.46 (-1.11 to 0.20)

*p < 0.05. ^a^Model 1 is adjusted for age (continuous), study center, working status and type of accommodation. ^b^Model 2 is adjusted for age (continuous), study center, smoking status, marital status, working status, education, type of accommodation, hours spent at home (continuous), having children, family history of obesity and exercise level

It appeared that larger traffic counts reported in the street nearby bedroom window corresponded to higher beta values for women and lower values for men (Table 3). According to the Model 1, women, who reported their bedroom window location nearby a street with much traffic had 2.30 (95% CI 0.47 to 4.13) cm higher WC compared to women who reported no bedroom window nearby street. For women, according to the Model 1, statistically significant associations were also found with moderate traffic for BMI and WC, but in fully adjusted models these associations disappeared. For men, there was no statistically significant associations between bedroom window location and obesity markers.

Women who had a bedroom window nearby the street and reported at the same time noise hearing in bedroom, had 0.28 (95% CI 0.03 to 0.53) kg/m^2^ higher BMI according to the fully adjusted model. According to the Model 1 among women also association between noise hearing in bedroom and WC was found, which was not statistically significant in fully adjusted model (Table 3). Associations for men were negative, although not statistically significant.

Similar associations for continuous obesity outcomes appeared also for binary outcomes. In the total population higher noise exposures reported in the bedroom (all three noise indicators) were associated with higher risks for being overweight (Table S1).

In stratified analysis, it appeared that women who reported higher exposure to road traffic noise in the bedroom had an increased risk of being overweight, obesity, and abdominal obesity with OR varying from 1.17 to 1.81 in models with minimal adjustment set (Table S1) and from 1.15 to 1.26 in fully adjusted models (Table 4). Risks increased with increasing exposure levels, although the differences were not always statistically significant influenced by a lower sample size in higher exposure levels. For men, the associations were again rather opposite. Men, who reported very much traffic noise in the bedroom, had a statistically significantly lower risk of abdominal obesity (Table 4).

**Table 4:** Associations between self-reported traffic noise in bedroom and binary obesity markers stratified by sex

		Overweight^a^	Obesity^b^	Abdominal obesity^c^
OR (95% CI)				
In your bedroom, can you hear traffic noise?	**Women** *(ref no)*			
	Little	1.21 (1.07 to 1.36)*	1.14 (0.96 to 1.35)	1.21 (1.07 to 1.37)*
	Much	1.22 (0.95 to 1.59)	1.25 (0.89 to 1.75)	1.20 (0.92 to 1.57)
	Very much	1.59 (0.94 to 2.70)	1.45 (0.78 to 2.59)	1.50(0.87 to 2.57)
	**Men** *(ref no)*			
	Little	0.97 (0.85 to 1.10)	0.98 (0.83 to 1.16)	0.88 (0.76 to 1.01)
	Much	0.85 (0.64 to 1.15)	0.97 (0.66 to 1.42)	0.75 (0.53 to 1.04)
	Very much	0.85 (0.44 to 1.67)	0.83 (0.33 to 2.05)	0.39 (0.17 to 0.91)*
Does your bedroom window face a nearby street (< 20 m)? Toward street with…	**Women** *(ref no)*			
	…little traffic	1.07 (0.95 to 1.21)	0.96 (0.81 to 1.15)	1.06 (0.93 to 1.21)
	… moderate traffic	1.26 (1.02 to 1.57)*	1.02 (0.76 to 1.37)	1.22 (0.98 to 1.52)
	… much traffic	0.99 (0.70 to 1.41)	1.49 (0.97 to 2.26)	1.26 (0.88 to 1.81)
	**Men** *(ref no)*			
	…little traffic	0.91 (0.79 to 1.05)	1.05 (0.87 to 1.25)	0.99 (0.85 to 1.14)
	… moderate traffic	1.10 (0.86 to 1.41)	0.77 (0.55 to 1.08)	0.80 (0.60 to 1.06)
	… much traffic	0.83 (0.57 to 1.24)	0.64 (0.33 to 1.22)	0.78 (0.49 to 1.26)
Participant reported at the same time bedroom window towards street and traffic noise hearing (no/yes)	**Women** *(ref no)*	1.21 (1.07 to 1.37)*	1.08 (0.91 to 1.28)	1.15 (1.02 to 1.31)*
	**Men** *(ref no)*	0.94 (0.82 to 1.08)	0.96 (0.81 to 1.15)	0.89 (0.77 to 1.04)

^a^BMI ≥ 25 kg/m^2^, ^b^BMI ≥ 30 kg/m^2^, ^c^women WC > 88 cm; men WC > 102 cm, *p < 0.05. Model is adjusted for: age (continuous), study center, smoking status, marital status, working status, education, dwelling type, hours spent at home (continuous), having obese parent (yes/no), having children (yes/no) and exercise level

We also investigated how the associations between noise hearing in the bedroom and BMI are modified by other factors such as air pollution, study center, and residential history. We used modelled NO_X_ levels at participant home addresses as air pollution indicator. In general NO_X_ levels were relatively low in all studied cities (mean 10.1, SD 5.6) ranging from min 0.5 to max 63.8 µg/m^3^ (Table S2). People who reported that they can hear more noise in their bedroom also had a higher exposure to NO_X_ (Fig. S5, Fig. S6). Adjustments for air pollution did not change the positive association of obesity markers with noise hearing markers. The stratified analysis showed that associations between noise hearing in bedroom and continuous obesity markers are smaller for women, who had low exposure to NOx, but it was the opposite for men. However, interactions were not statistically significant (Fig. S7, S8, S9, S10).

The associations among women between continuous obesity markers and self-reported traffic noise in bedroom were not statistically significant in subgroups of study centers (Fig. S7, S8). For men significant heterogeneity between study centers was observed. In Swedish cities (Gothenburg, Uppsala, Umea), the associations were negative, but in Tartu and Reykjavik positive. For both genders, the population restriction for only those who have lived at the same address for at least 10 years, did not change the results.

We assessed the mediating effect of sleep disturbance on the relationship between noise exposure in bedroom and continuous obesity markers among women. The results revealed a significant partial or full mediation effect, depending on obesity marker used. The indirect effect of noise via sleep disturbance is positive and statistically significant for all noise and obesity indicators used indicating significant mediation by sleep disturbance (Table 5). Noise in presence of sleep disturbance in the model (direct effect) has significant positive effect in most cases for BMI, but not in case for WC. It means, that in the case of BMI we size mostly partial mediation (except first analysis) and in case of WC full mediation of sleep disturbance in the association between noise and obesity indicators.

**Table 5:** Mediation analysis, noise reporting in bedroom and obesity indicators trough sleep disturbance score among women

		BMI	WC
		Beta (95% CI)	Beta (95% CI)
In your bedroom, can you hear traffic noise? (no/yes)	Direct effect	0.27 (-0.01 to 0.54)	0.34 (-0.39 to 1.079
	Indirect effect^a^	0.03 (0.01 to 0.06)*	1.12 (0.04 to 0.21)*
	Total effect	0.30 (0.02 to 0.58)*	0.46 (-0.27 to 1.19)
Does your bedroom window face a nearby street (< 20 m)? (no/yes)	Direct effect	0.31 (0.03 to 0.59)*	0.43 (-0.30 to 1.169
	Indirect effect	0.02 (0.00 to 0.04)*	0.08 (0.01 to 0.169*
	Total effect	0.33 (0.05 to 0.61)*	0.50 (-0.23 to 1.24)
Participant reported at the same time bedroom window towards street and traffic noise hearing (no/yes)	Direct effect	0.15 (0.03 to 0.62)*	0.40 (-0.36 to 1.17)
	Indirect effect	0.03 (0.01 to 0.06)*	0.11 (0.03 to 0.21)*
	Total effect	0.35 (0.06 to 0.65)*	0.51 (-0.25 to 1.28)

*p < 0.05. As covariates were included to the model: age (continuous), study center, working status and type of accommodation. ^a^Percentile bootstrap estimate of CI

## Discussion

Our results confirm the positive association between traffic noise and markers of obesity that have been previously shown by number of studies [9, 11, 23, 24, 29, 30, 35–39]. In the present study we found clear positive relationships between perceived traffic noise in the bedroom and all used obesity markers (WC, BMI, being overweight, obesity, and abdominal obesity), however, only for women. For men, there were no associations, or they were rather opposite. These results are like the findings of Li et al. (2021), who measured nocturnal traffic noise in the bedroom for a week, and found that higher noise exposure increases the BMI among women, but not among men. Furthermore, several other previous studies with different noise exposure metrics (perceived or modelled) have found statistically significant associations between traffic noise and different obesity markers, mainly among women [23, 26, 30, 35, 39]. Further research should be undertaken to confirm the possible important effect modification by sex and to find a pathophysiological explanation for this phenomenon.

The results of our study demonstrate the importance of nocturnal traffic noise in the development of obesity or being overweight. The mediation analysis clearly indicated that sleep disturbance partially or fully mediates the association between noise in bedroom and obesity markers among women. It has been suggested that the intermittent nature of nocturnal road traffic is disturbing to sleep continuity [40]. It can lead to sleep disturbance and deprivation, which in turn can lead to obesity [19–22]. Earlier studies in the same RHINE III cohort found that traffic noise was strongly associated with insomnia symptoms (difficulty initiating sleep, difficulty maintaining sleep, and with early morning awakenings) [41] and daytime sleepiness and habitual snoring [42]. Basner et al. (2014) has shown that nocturnal noise exposure can lead to obesity without perceived sleep disturbance through stress reactions, as a stress response during sleep has been observed starting from very low noise levels and chronic stress has been suggested to contribute to obesity [18].

One of the main limitations of the current study is the self-reported noise exposure and obesity data, that might have resulted in some reporting biases. For instance, in study of [43], the reported road traffic noise annoyance was compared with modelled exposure and only a fair association between them was found (Spearman correlation *r*_s_ = 0.37). However, noise modelling is usually done for the street-side façade of the building at 4 m height, not considering the specific location of each respondent in a building (e.g., street vs. back-yard side, exact floor etc.)[44]. In present study we did not ask if the participant is annoyed/disturbed by traffic noise, but just if he/she can hear it or the bedroom window is located nearby street. This way of forming the question may have reduced the reporting biases.

However, the reporting of hearing noise in the bedroom can be affected by the subjective noise sensitivity of participants. Previous studies have shown that for similar exposure levels, noise sensitive individuals tend to report more annoyance than non-sensitive individuals [45]. Taking this fact into account, self-reported noise measures could be a better indicator for annoyance among noise-sensitive people than purely modelled levels [46]. In the current study, there was no evaluation of the participant’s noise sensitivity, but the interpretation of answers to two different questions can give some information about noise sensitivity. In general, betas and ORs were higher for women who reported noise hearing in the bedroom compared to women who reported a bedroom location nearby a street or reported both noise exposure metrics at the same time. This could reflect the possible effect modification of noise sensitivity, which is consistent with the findings of Oftedal et al. (2015), who found associations between modeled levels of road traffic noise and obesity markers (BMI and WC) only among noise sensitive women. In this light, the findings of present study – that show an increase of WC by 3.30 cm and BMI by 1.30 kg/m^2^ if there is very much traffic noise in bedroom – are more relevant for noise sensitive women. In the present analysis with a more objective noise metric, window location nearby a street, the increase of WC was lower. In the analysis where we used a combined noise indicator, the associations were smallest (WC by 0.64 cm and BMI by 0.28 kg/m^2^). We assume that the combined variable describes most realistic exposure situation in participants bedroom as it reduces over-reporting. Nnevertheless, current findings suggest that noise exposure might be a risk factor for obesity also among non-sensitive women, but noise sensitivity can be an important effect modifier that increases the effect of road traffic noise.

Most of the studies that have examined the associations between traffic noise and markers of obesity, have used modeled noise levels at the most exposed façade of the buildings [23, 26, 35, 37, 38], but as discussed earlier, the exposure assessment created using indoor self-reported data can also provide some advantages over modeled traffic noise exposure assessments. Studies with modeled noise levels have objective data for time-average outdoor traffic noise, but it remains unclear how much all participants can actually hear the noise, as it depends from noise insulation of the building, in which floor the participant lives, the bedroom’s location toward a street, exposure time etc. For example, in Norway facade insulation reduced the proportion of respondents highly annoyed by traffic noise from 43 to 15% [47]. Self-reported data, although subjective, can give some information about indoor noise levels at each participant’s home and, therefore, confirm the evidence found in studies with modelled noise data.

To reduce the reporting bias of obesity markers, a measuring tape was sent to the participants together with the questionnaire. However, many studies have shown, that study participants tend to underreport their body weight and overreport body height, although some studies find opposite associations [48]This trend is similar for both women and men, although men tend to more overestimate their height than women [49]. Self-measured WC is also shown to be underestimated [50] and overestimated [51] For both genders, the most accurate BMI values based on self-reported height and weight are obtained between the ages 42 and 55 [49]. RHINE cohort in current study was in age 38 to 66. Despite the inaccuracies associated with the self-reported obesity markers, they are still considered to be a useful tool for large-scale epidemiological studies and have been widely used [49]. Participants, who are exposed to higher levels of road traffic noise, are at the same time also exposed to higher levels of air pollution from road traffic as the source of both pollutions is the same. Presently, we used modelled NO_X_ concentrations as traffic induced air pollution markers at the home addresses of participants. NO_X_ has been shown to be an effective marker of local traffic pollution [52]. NOx is widely used in other studies focusing to traffic noise associations with obesity markers [23, 26, 35, 36, 39]. We saw higher than average NO_X_ concentrations among participants who reported more noise in bedroom or bedroom window towards street with more traffic (Fig. S5 and S6). This association could be the indicator of the respective quality of noise reporting in the present study, as higher modeled NO_X_ indicates a higher density of road traffic and should be correlated with higher noise levels from road traffic.

Our results suggest that road traffic noise has an effect on obesity/being overweight, independent of air pollution. In line with other studies [23, 26, 35, 36, 39], adjustments for the air quality indicator NO_X_ did not change the positive association between noise exposure metrics and obesity markers. To our knowledge, there is no epidemiological evidence that associated the NO_X_ with obesity in humans, but recently published studies indicate that another air quality indicator, particulate matter, could increase the risk of being overweight [53] and also have a confounding role in association between traffic noise and obesity [36]. In the analyses of this paper we did not have available data on particulate matter, but this could be included in future studies.

Another limitation of our study is that we could not adjust for alcohol consumption, diet, income, ethnicity and mental status, which might have contributed to the risk of obesity. However, we were able to adjust for many other potential confounding factors, including physical activity, working status and education (that also indicate socioeconomic status), family history of obesity as a surrogate measure for genetic inheritance/familial disposition, type of accommodation (detached house, terraced house or an apartment), and hours spent at home – data which are mostly missing in other studies.

A strength of the study was its large multicenter randomly selected study population, which allowed us to also run stratified analysis. We found the evidence that sleep disturbance mediates the impact of road traffic noise to the obesity which has not been well studied so far. Nevertheless, as this is a cross-sectional study, a causal relationship cannot be confirmed.

## Conclusion

The findings in this study support the hypothesis that higher exposure to nocturnal indoor road traffic noise increases the risk of being overweight or obese, but only among women. Sleep disturbance partially or fully mediates the association between noise in bedroom and obesity markers among women. For men, there were no clear associations or even the opposite association. Among women all studied obesity markers (WC, BMI, being overweight, obesity, and abdominal obesity) were positively associated with the self-reported noise hearing level in the bedroom and with the bedroom window’s location towards the street. Further research using longitudinal analysis, should be undertaken to confirm our findings and to examine the possible important effect modification by sex.

## Electronic supplementary material

Below is the link to the electronic supplementary material.

**Fig. S1** Directed acyclic graph for the variable selection in the adjusted models SES ? socioeconomic status. **Fig. S2** Mean of BMI by study center (95% CI). **Fig. S3** Mean waist circumference of women by study center (95% CI). **Fig. S4** Mean waist circumference of men by study center (95% CI). **Fig. S5** Self-reported traffic noise in bedroom in relation with modelled mean NOx μg/m3. Answers to the question: “Does your bedroom window face a nearby street (< 20 m)? 1?no traffic, 2? a street with little traffic, 3 ?a street with moderate traffic, 4?a street with much traffic”. **Fig. S6** Self-reported traffic noise in bedroom in relation with modelled mean NOx μg/m3. Answers to the question: “In your bedroom, can you hear traffic noise? 1?not at all, 2?a little, 3?a great deal, 4?very much”. **Table S1**. Associations between self-reported noise in bedroom and binary obesity markers. **Table S2**. Mean annual average NOX concentrations at participant home addresses (μg/m3). **Fig. S7** Associations (betas with 95% CI) between self-reported traffic noise in the bedroom (no, yes) and BMI among women stratified by, air pollution group, study centers, residential history, type of accommodation, hours spent at home and exercise level. Model is adjusted for age (continuous), study center, smoking status, marital status, working status, education, type of accommodation, hours spent at home (continuous), having children, family history of obesity and exercise level. **Fig. S8** Associations (betas with 95% CI) between self-reported traffic noise in the bedroom (no, yes) and waist circumference among women stratified by air pollution group, study centers, residential history, type of accommodation, hours spent at home and exercise level. Model is adjusted for age (continuous), study center, smoking status, marital status, working status, education, type of accommodation, hours spent at home (continuous), having children, family history of obesity and exercise level. **Fig. S9** Associations (betas with 95% CI) between self-reported traffic noise in the bedroom (no, yes) and BMI among men stratified by air pollution group, study centers, residential history, type of accommodation, hours spent at home and exercise level. Model is adjusted for age (continuous), study center, smoking status, marital status, working status, education, type of accommodation, hours spent at home (continuous), having children, family history of obesity and exercise level. **Fig. S10** Associations (betas with 95% CI) between self-reported traffic noise in the bedroom (no, yes) and waist circumference among men stratified by air pollution group, study centers, residential history, type of accommodation, hours spent at home and exercise level. Model is adjusted for age (continuous), study center, smoking status, marital status, working status, education, type of accommodation, hours spent at home (continuous), having children, family history of obesity and exercise level.

## Data Availability

The datasets analyzed during the current study are not publicly available because individual privacy could be compromised, but are available from the corresponding author on reasonable request with permission of ethics committee of the respective country.

## List of abbreviations

BMI: Body mass index
DAG: Directed Acyclic Graph
dB: Decibels
HPA: Hypothalamic–pituitary–adrenocortical
L_Amax_: Maximum A-weighted noise level during a particular measurement
NO_X_: Nitrogen oxides
OR: Odds ratio
RHINE: Respiratory Health in Northern Europe
SD: Standard deviation
VIF: Variance inflation factor
WC: Waist circumference
WHO: World Health Organization
