# Supplementary material for: Traffic noise in the bedroom in association with markers of obesity: a cross-sectional study and mediation analysis of the respiratory health in Northern Europe cohort
Source: BMC Public Health. 2023 Jun 27;23:1246. doi: 10.1186/s12889-023-16128-2 (PMC10294471; doi:10.1186/s12889-023-16128-2)
Supplement: Supplementary file 1 — Fig. S1 Directed acyclic graph for the variable selection in the adjusted models SES ? socioeconomic status. Fig. S2 Mean of BMI by study center (95% CI). Fig. S3 Mean waist circumference of women by study center (95% CI). Fig. S4 Mean waist circumference of men by study center (95% CI). Fig. S5 Self-reported traffic noise in bedroom in relation with modelled mean NOx μg/m3. Answers to the question: “Does your bedroom window face a nearby street (< 20 m)? 1?no traffic, 2? a street with little traffic, 3 ?a street with moderate traffic, 4?a street with much traffic”. Fig. S6 Self-reported traffic noise in bedroom in relation with modelled mean NOx μg/m3. Answers to the question: “In your bedroom, can you hear traffic noise? 1?not at all, 2?a little, 3?a great deal, 4?very much”. Table S1. Associations between self-reported noise in bedroom and binary obesity markers. Table S2. Mean annual average NOX concentrations at participant home addresses (μg/m3). Fig. S7 Associations (betas with 95% CI) between self-reported traffic noise in the bedroom (no, yes) and BMI among women stratified by, air pollution group, study centers, residential history, type of accommodation, hours spent at home and exercise level. Model is adjusted for age (continuous), study center, smoking status, marital status, working status, education, type of accommodation, hours spent at home (continuous), having children, family history of obesity and exercise level. Fig. S8 Associations (betas with 95% CI) between self-reported traffic noise in the bedroom (no, yes) and waist circumference among women stratified by air pollution group, study centers, residential history, type of accommodation, hours spent at home and exercise level. Model is adjusted for age (continuous), study center, smoking status, marital status, working status, education, type of accommodation, hours spent at home (continuous), having children, family history of obesity and exercise level. Fig. S9 Associations (betas with 95% C [file 12889_2023_16128_MOESM1_ESM.docx]

**Supplementary material**

**Traffic noise in the bedroom in association with markers of obesity: a cross-sectional study and mediation analysis of the Respiratory Health in Northern Europe cohort**

Triin Veber^a^, Andrei Pyko^b,c^, Hanne Krage Carlsen^d^, Mathias Holm^d^*,* Thorarinn Gislason^e^*,* Christer Janson^f^, Ane Johannessen^g^*,* Johan Nilsson Sommar^h^ , Lars Modig^h^, Eva Lindberg^f^*,* Vivi Schlünssen^i^*,* Karolin Toompere^a^*,* Hans Orru^a,j*^

***Corresponding author: Hans Orru**

Section of Sustainable Health, Department of Public Health and Clinical Medicine, Umeå University, Umeå, Sweden

Institute of Family Medicine and Public Health, University of Tartu, Tartu, Estonia

e-mail address: Hans.Orru@umu.se


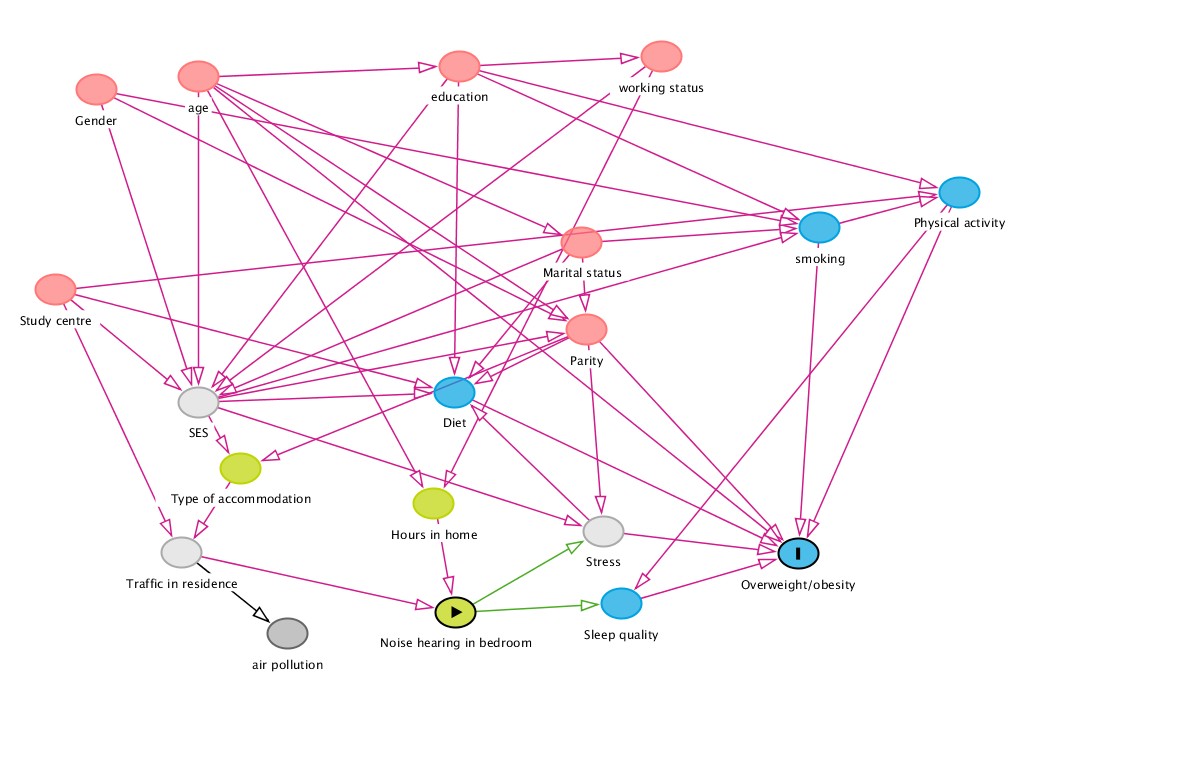

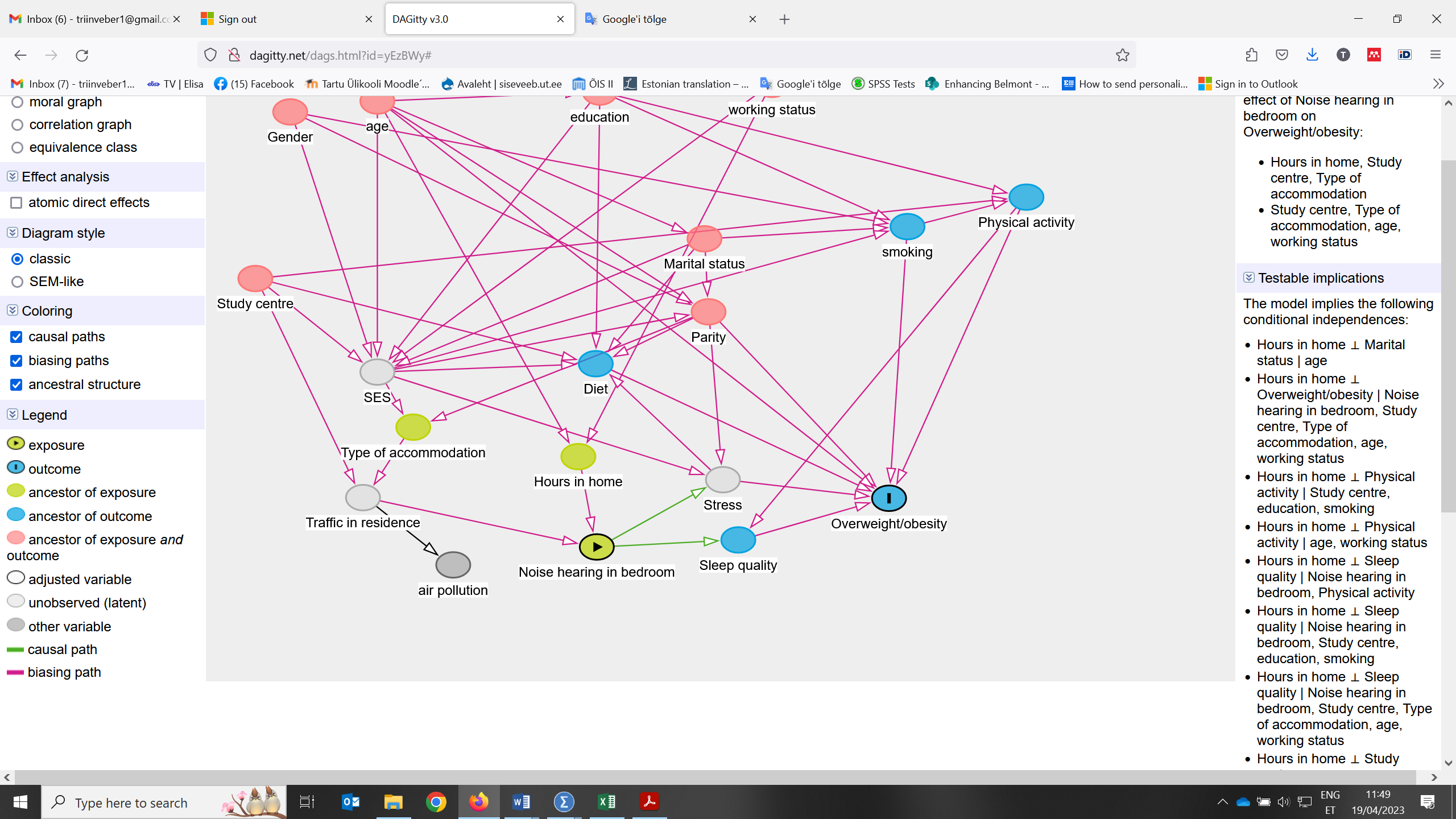


**Fig. S1** Directed acyclic graph for the variable selection in the adjusted models

SES – socioeconomic status


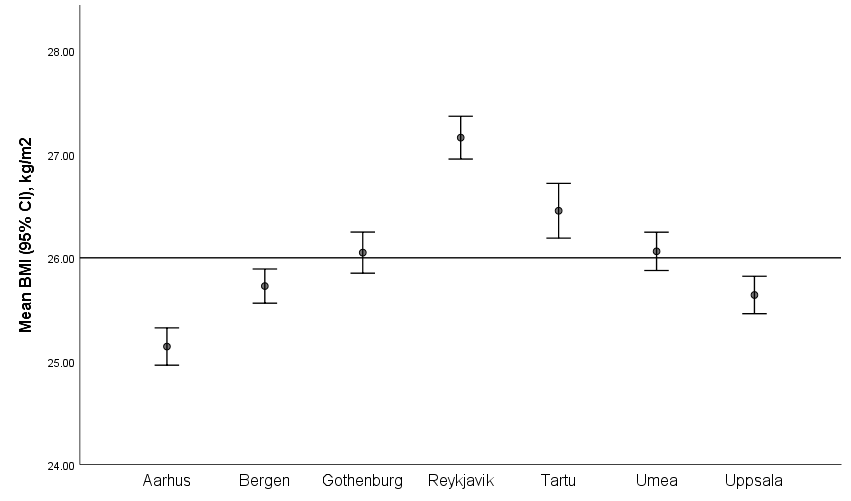


**Fig. S2** Mean of BMI by study center (95% CI)


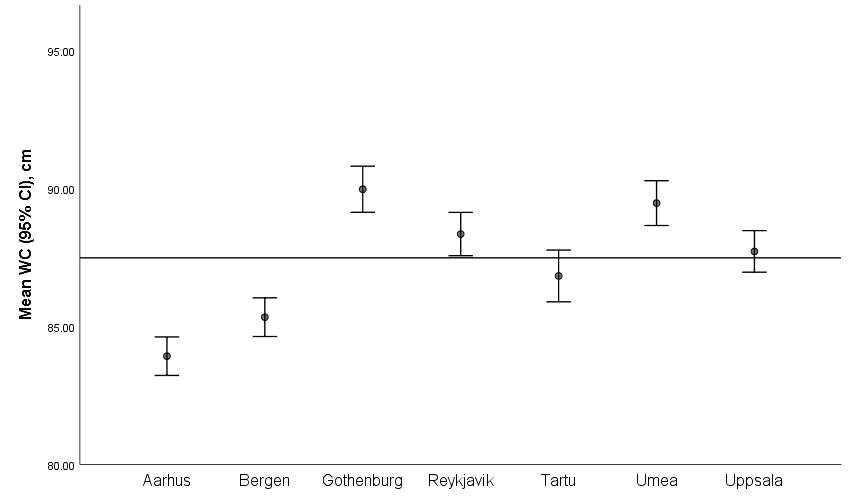


**Fig. S3** Mean waist circumference of women by study center (95% CI)


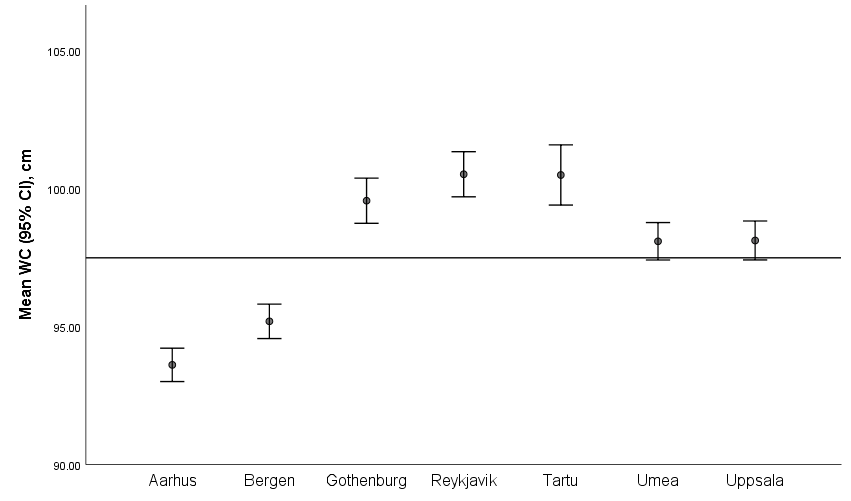


**Fig. S4** Mean waist circumference of men by study center (95% CI)


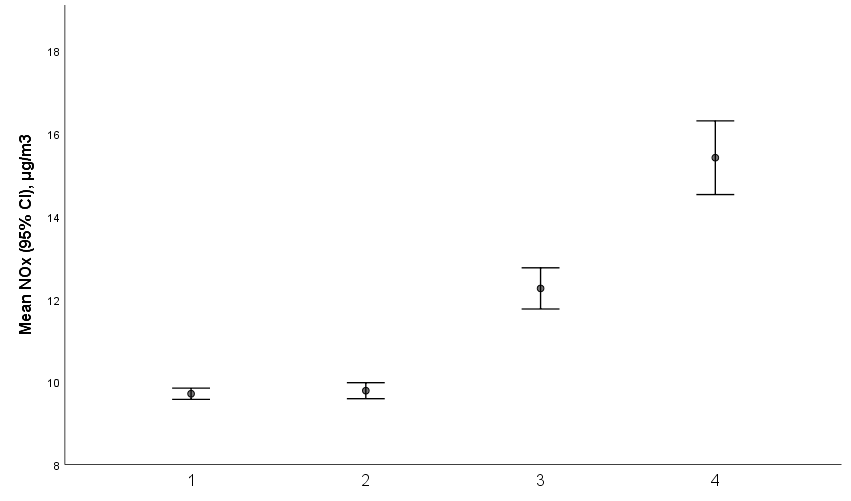


**Fig. S5** Self-reported traffic noise in bedroom in relation with modelled mean NOx μg/m^3^. Answers to the question: “Does your bedroom window face a nearby street (< 20 m)? 1–no traffic, 2– a street with little traffic, 3 –a street with moderate traffic, 4–a street with much traffic”


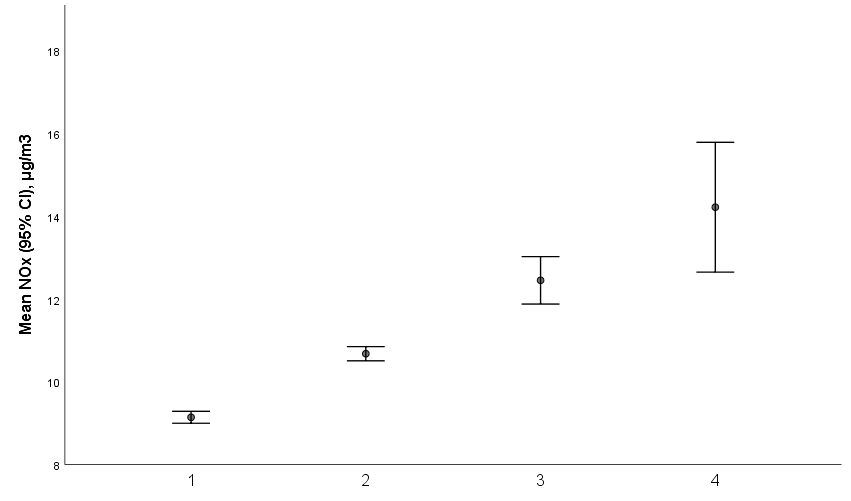


**Fig. S6** Self-reported traffic noise in bedroom in relation with modelled mean NOx μg/m^3.^ Answers to the question: “In your bedroom, can you hear traffic noise? 1–not at all, 2–a little, 3–a great deal, 4–very much”

**Table S1**. Associations between self-reported noise in bedroom and binary obesity markers

|  |  | Model | Overweight^a^ | | Obesity^b^ | | Abdominal obesity^c^ | |
| --- | --- | --- | --- | --- | --- | --- | --- | --- |
|  |  |  | N | OR (95% CI) | N | OR (95% CI) | N | OR (95% CI) |
| In your bedroom, can you hear traffic noise? | All | **Model 1** *(ref no)* | 5998 |  | 6047 |  | NA^f^ | NA |
|  |  | Little | 5568 | 1.09 (1.01 to 1.17)* | 5623 | 1.09 (0.99 to 1.21) | NA | NA |
|  |  | Much | 667 | 1.19 (0.92 to 1.23) | 672 | 1.04 (0.83 to 1.29) | NA | NA |
|  |  | Very much | 131 | 1.32 (0.92 to 1.91) | 133 | 1.30 (0.85 to 1.99) | NA | NA |
|  |  | **Model 2** *(ref no)* | 5092 |  | 5130 |  | NA | NA |
|  |  | Little | 4611 | 1.09 (0.99 to 1.19) | 4660 | 1.06 (0.94 to 1.20) | NA | NA |
|  |  | Much | 533 | 1.05 (0.86 to 1.27) | 538 | 1.10 (0.86 to 1.41) | NA | NA |
|  |  | Very much | 107 | 1.24 (0.82 to 1.88) | 108 | 1.14 (0.70 to 1.86) | NA | NA |
|  | F^d^ | **Model 1** *(ref no)* | 3082 |  | 3119 |  | 2881 |  |
|  |  | Little | 3018 | 1.17 (1.06 to 1.30)* | 3056 | 1.17 (1.01 to 1.36)* | 2876 | 1.18 (0.06 to 1.32)* |
|  |  | Much | 380 | 1.25 (1.00 to 1.56)* | 385 | 1.19 (0.89 to 1.59) | 358 | 1.21 (0.96 to 1.52) |
|  |  | Very much | 80 | 1.68 (1.07 to 2.66)* | 85 | 1.63 (0.97 to 2.73) | 75 | 1.81 (1.12 to 2.92)* |
|  |  | **Model 2** *(ref no)* | 2585 |  | 2613 |  | 2440 |  |
|  |  | Little | 2451 | 1.21 (1.07 to 1.36)* | 2486 | 1.14 (0.96 to 1.35) | 2350 | 1.21 (1.07 to 1.37)* |
|  |  | Much | 304 | 1.22 (0.95 to 1.59) | 309 | 1.25 (0.89 to 1.75) | 287 | 1.200 (0.92 to 1.57) |
|  |  | Very much | 66 | 1.59 (0.94 to 2.70) | 67 | 1.45 (0.78 to 2.59) | 62 | 1.50 (0.87 to 2.57) |
|  | M^e^ | **Model 1** *(ref no)* | 2916 |  | 2928 |  | 2722 |  |
|  |  | Little | 2550 | 0.99 (0.89 to 1.12) | 2567 | 1.02 (0.88 to 1.18) | 2408 | 0.91 (0.80 to 1.03) |
|  |  | Much | 287 | 0.89 (0.69 to 1.16) | 287 | 0.90 (0.64 to 1.26) | 265 | 0.74 (0.55 to 0.99)* |
|  |  | Very much | 48 | 0.87 (0.48 to 1.58) | 48 | 1.93 (0.43 to 2.04) | 46 | 0.45 (0.22 to 0.96)* |
|  |  | **Model 2** *(ref no)* | 2507 |  | 2517 |  | 2357 |  |
|  |  | Little | 2160 | 0.97 (0.85 to 1.10) | 2174 | 0.98 (0.83 to 1.16) | 2050 | 0.88 (0.76 to 1.01) |
|  |  | Much | 229 | 0.85 (0.64 to 1.15) | 229 | 0.97 (0.66 to 1.42) | 211 | 0.75 (0.53 to 1.04) |
|  |  | Very much | 41 | 0.85 (0.44 to 1.67) | 41 | 0.83 (0.33 to 2.05) | 40 | 0.39 (0.17 to 0.91) |
| Does your bedroom window face a nearby street (< 20 m)? (Toward street with little traffic/ moderate traffic/much traffic) | All | **Model 1** *(ref no)* | 7052 |  | 7124 |  | NA | NA |
|  |  | Little | 4031 | 1.02 (0.94 to 1.10) | 4064 | 1.04 (0.93 to 1.56) | NA | NA |
|  |  | Moderate | 946 | 1.22 (1.06 to 1.41)* | 953 | 0.94 (0.77 to 1.14) | NA | NA |
|  |  | Much | 329 | 0.94 (0.75 to 1.19) | 328 | 1.05 (0.76 to 1.42) | NA | NA |
|  |  | **Model 2** *(ref no)* | 5922 |  | 5983 |  | NA | NA |
|  |  | Little | 3367 | 0.99 (0.90 to 1.07) | 3392 | 1.01 (0.89 to 1.14) | NA | NA |
|  |  | Moderate | 784 | 1.19 (1.01 to 1.39)* | 791 | 0.91 (0.73 to 1.14) | NA | NA |
|  |  | Much | 262 | 0.92 (0.71 to 1.20) | 262 | 1.09 (0.77 to 1.53) | NA | NA |
|  | F | **Model 1** *(ref no)* | 3672 |  | 3719 |  | 3482 |  |
|  |  | Little | 2170 | 1.08 (0.97 to 1.21) | 2196 | 1.02 (0.88 to 1.19) | 2036 | 1.08 (0.97 to 1.21) |
|  |  | Moderate | 522 | 1.30 (1.08 to 1.58)* | 530 | 1.11 (0.87 to 1.43) | 492 | 1.26 (1.03 to 1.53)* |
|  |  | Much | 195 | 1.05 (0.78 to 1.41) | 196 | 1.45 (1.01 to 2.08)* | 176 | 1.34 (0.98 to 1.82) |
|  |  | **Model 2** *(ref no)* | 3044 |  | 3085 |  | 2908 |  |
|  |  | Little | 1779 | 1.07 (0.95 to 1.21) | 1799 | 0.99 (0.81 to 1.15) | 1679 | 1.06 (0.93 to 1.21) |
|  |  | Moderate | 428 | 1.26 (1.02 to 1.57)* | 435 | 1.02 (0.76 to 1.37) | 409 | 1.22 (0.98 to 1.52) |
|  |  | Much | 151 | 0.99 (0.70 to 1.41) | 152 | 1.49 (0.97 to 2.26) | 140 | 1.26 (0.88 to 1.81) |
|  | M | **Model 1** *(ref no)* | 3380 |  | 3405 |  | 3186 |  |
|  |  | Little | 1861 | 0.94 (0.83 to 1.6) | 1868 | 1.05 (0.90 to 1.23) | 1746 | 0.96 (0.84 to 1.10) |
|  |  | Moderate | 424 | 1.13 (0.90 to 1.40) | 423 | 0.76 (0.56 to 1.03) | 384 | 0.78 (0.61 to 0.99)* |
|  |  | Much | 134 | 0.84 (0.59 to 1.21) | 132 | 0.60 (0.34 to 1.06) | 125 | 0.73 (0.48 to 1.11) |
|  |  | **Model 2** *(ref no)* | 2878 |  | 2898 |  | 2724 |  |
|  |  | Little | 1588 | 0.91 (0.79 to 1.05) | 1593 | 1.05 (0.87 to 1.25) | 1507 | 0.99 (0.85 to 1.14) |
|  |  | Moderate | 356 | 1.10 (0.86 to 1.41) | 356 | 0.77 (0.55 to 1.08) | 320 | 0.80 (0.60 to 1.06) |
|  |  | Much | 111 | 0.83 (0.57 to 1.24) | 110 | 0.64 (0.33 to 1.22) | 104 | 0.78 (0.49 to 1.26) |
| Participant reported at the same time bedroom window towards street and traffic noise hearing (yes/no) | All | **Model 1** *(ref no)* | 8574 |  | 8658 |  |  |  |
|  |  | **Yes** | 3772 | 1.10 (1.01 to 1.19)* | 3799 | 1.05 (0.95 to 1.17) |  | NA |
|  |  | **Model 2** *(ref no)* | 7224 |  | 7292 |  |  |  |
|  |  | **yes** | 3104 | 1.08 (0.99 to 1.18) | 3129 | 1.03 (0.92 to 1.16) |  | NA |
|  | F | **Model 1** *(ref no)* | 4461 |  | 4519 |  | 4202 |  |
|  |  | **yes** | 2091 | 1.20 (1.08 to 1.34)* | 2115 | 1.14 (0.99 to 1.32) | 1978 | 1.18 (1.06 to 1.32)* |
|  |  | **Model 2** *(ref no)* | 3703 |  | 3750 |  | 3518 |  |
|  |  | **yes** | 1694 | 1.21 (1.07 to 1.37)* | 1716 | 1.08 (0.91 to 1.28) | 1613 | 1.15 (1.02 to 1.31)* |
|  | M | **Model 1** *(ref no)* | 4113 |  | 4139 |  | 3866 |  |
|  |  | **yes** | 1681 | 0.98 (0.86 to 1.10) | 1684 | 0.96 (0.83 to 1.13) | 1571 | 0.89 (0.78 to 1.02) |
|  |  | **Model 2** *(ref no)* | 3521 |  | 3542 |  | 3327 |  |
|  |  | **yes** | 1410 | 0.94 (0.82 to 1.08) | 1413 | 0.96 (0.81 to 1.15) | 1327 | 0.89 (0.77 to 1.04) |

^a^BMI ≥ 25 kg/m^2^; ^b^BMI ≥ 30 kg/m^2^; ^c^women WC>88 cm, men WC>102 cm; ^d^F–Female; ^e^M–Male; ^f^NA- not applicable. *p<0.05. Model 1 is minimal sufficient adjustment set suggested by DAGitty adjusted for sex, age (continues), study center, working status, type of accommodation.. Model 2 is fully adjusted model adjusted for sex, age (continuous), study center, smoking status, marital status, working status, education, type of accommodation, hours spent at home (continuous), family history of obesity, exercise level and having children.

**Table S2**. Mean annual average NO_X_ concentrations at participant home addresses (μg/m^3^)

|  | Mean (±SD) | Minimum | Maximum |
| --- | --- | --- | --- |
| Aarhus | 9.9 (±1.2) | 8.8 | 16.1 |
| Bergen | 11.9 (±6.4) | 2.1 | 63.8 |
| Gothenburg | 16.0 (±7.0) | 4.1 | 54.9 |
| Reykjavik | 6.6 (±3.8) | 0.5 | 19.9 |
| Tartu | 13.0 (±6.3) | 2.7 | 54.3 |
| Umea | 13.8 (±5.9) | 2.8 | 46.8 |
| Uppsala | 6.5 (±2.9) | 3.3 | 24.2 |

**Fig. S7** Associations (betas with 95% CI) between self-reported traffic noise in the bedroom (no, yes) and BMI among women stratified by, air pollution group, study centers, residential history, type of accommodation, hours spent at home and exercise level. Model is adjusted for age (continuous), study center, smoking status, marital status, working status, education, type of accommodation, hours spent at home (continuous), having children, family history of obesity and exercise level.

**Fig. S8** Associations (betas with 95% CI) between self-reported traffic noise in the bedroom (no, yes) and waist circumference among women stratified by air pollution group, study centers, residential history, type of accommodation, hours spent at home and exercise level. Model is adjusted for age (continuous), study center, smoking status, marital status, working status, education, type of accommodation, hours spent at home (continuous), having children, family history of obesity and exercise level.

**Fig. S9** Associations (betas with 95% CI) between self-reported traffic noise in the bedroom (no, yes) and BMI among men stratified by air pollution group, study centers, residential history, type of accommodation, hours spent at home and exercise level. Model is adjusted for age (continuous), study center, smoking status, marital status, working status, education, type of accommodation, hours spent at home (continuous), having children, family history of obesity and exercise level.

**Fig. S10** Associations (betas with 95% CI) between self-reported traffic noise in the bedroom (no, yes) and waist circumference among men stratified by air pollution group, study centers, residential history, type of accommodation, hours spent at home and exercise level. Model is adjusted for age (continuous), study center, smoking status, marital status, working status, education, type of accommodation, hours spent at home (continuous), having children, family history of obesity and exercise level.
